# Assessing Risk in Implementing New Artificial Intelligence Triage Tools—How Much Risk is Reasonable in an Already Risky World?

**DOI:** 10.1007/s41649-024-00348-8

**Published:** 2025-01-29

**Authors:** Alexa Nord-Bronzyk, Julian Savulescu, Angela Ballantyne, Annette Braunack-Mayer, Pavitra Krishnaswamy, Tamra Lysaght, Marcus E. H. Ong, Nan Liu, Jerry Menikoff, Mayli Mertens, Michael Dunn

**Affiliations:** 1 Centre for Biomedical Ethics, National University of Singapore, Singapore; 2 University of Otago, Wellington, New Zealand; 3 School of Health and Society, University of Wollongong, Wollongong, Australia; 4 Institute for Infocomm Research, Agency for Science, Technology and Research (A*STAR), Singapore; 5 Sydney Health Ethics, Faculty of Medicine and Health, University of Sydney, Sydney, Australia; 6 Center for Quantitative Medicine, Duke-NUS Medical School, Singapore; 7 Department of Philosophy, University of Antwerp, Antwerp, Belgium; 8 Program in Health Services and Systems Research, Duke-NUS Medical School, Singapore; 9 Department of Emergency Medicine, Singapore General Hospital, Singapore; 10 NUS Artificial Intelligence Institute, National University of Singapore, Singapore; 11 Uehiro Oxford Institute, University of Oxford, Oxford, UK

**Keywords:** Artificial intelligence, Triage, Implementation, Risk

## Abstract

Risk prediction in emergency medicine (EM) holds unique challenges due to issues surrounding urgency, blurry research-practise distinctions, and the high-pressure environment in emergency departments (ED). Artificial intelligence (AI) risk prediction tools have been developed with the aim of streamlining triaging processes and mitigating perennial issues affecting EDs globally, such as overcrowding and delays. The implementation of these tools is complicated by the potential risks associated with over-triage and under-triage, untraceable false positives, as well as the potential for the biases of healthcare professionals toward technology leading to the incorrect usage of such tools. This paper explores risk surrounding these issues in an analysis of a case study involving a machine learning triage tool called the Score for Emergency Risk Prediction (SERP) in Singapore. This tool is used for estimating mortality risk in presentation at the ED. After two successful retrospective studies demonstrating SERP’s strong predictive accuracy, researchers decided that the pre-implementation randomised controlled trial (RCT) would not be feasible due to how the tool interacts with clinical judgement, complicating the blinded arm of the trial. This led them to consider other methods of testing SERP’s real-world capabilities, such as ongoing-evaluation type studies. We discuss the outcomes of a risk–benefit analysis to argue that the proposed implementation strategy is ethically appropriate and aligns with improvement-focused and systemic approaches to implementation, especially the learning health systems framework (LHS) to ensure safety, efficacy, and ongoing learning.

## Introduction

Risk prediction in emergency medicine (EM) is challenging and exacerbated by the complexity of patient factors, along with external factors such as environmental and system factors. According to the European Parliament,

Risk prediction focuses on assessing the likelihood of individuals experiencing a specific health condition or outcome. It typically generates probabilities for a wide array of outcomes ranging from death to adverse disease events (e.g. stroke, myocardial infarction, bone fracture). The process involves the identification of individuals with certain diseases or conditions and their classification according to stage, severity, and other characteristics. These individuals may subsequently be targeted to receive specific medical interventions (Lekadir et al. 2022).

Emergency departments (ED s) face unique challenges when it comes to risk prediction; the stakes are high, timing is critical, and resources are often limited. Triage nurses are specially trained to identify patients’ risk levels and determine patient prioritisation based on various factors such as vital signs, level of pain, and past medical history. With other external factors such as overcrowding, staffing issues, and limited resources at play, triage nurses are often faced with an especially complicated task. As a result, concerns have been raised about the quality of care in EDs globally when the system is under strain, including in Singapore, from which we will draw on a local case study as the focus of this paper (Fong et al. 2018, Elkum et al. 2011).

Artificial intelligence (AI) risk prediction tools aim to help EDs more easily and efficiently triage patients, making wait times shorter and patient prioritisation more accurate. However, they also raise ethical concerns that are exacerbated by their use in a high stakes environment and their relationship with clinical judgement. Although traditional clinical decision tools have long been available, there have been questions about their value in the clinical setting stemming from worries about limited predictive accuracy (Lekadir et al. 2022). The use of AI poses new opportunities to improve the accuracy of clinical decision tools.

In Singapore, there have been recent examples demonstrating these improvements. The case study driving this paper concerns the use of the Score for Emergency Risk Prediction (SERP), a machine learning triage tool used for estimating mortality after emergency admissions. RapidAI is another example of a successful risk prediction tool whereby stroke patients are identified in less than a minute, thus shaving off precious minutes of response time (National University Hospital 2023).

In order to implement SERP, a randomised controlled trial (RCT) would be the usual route to validate effectiveness and safety in clinical practise. As the title indicates, an RCT involves randomisation to balance participant characteristics between the intervention group—the group to receive the new treatment or intervention—and the comparator group—otherwise known as the ‘control’ group, or the group not receiving the new intervention. Participants are carefully selected and randomly assigned to one group so that any differences in outcomes between groups can clearly be attributed to the study intervention. RCTs are also often blinded thus further reducing biases and rigorously examining cause-effect relationships between intervention and outcome.

However, RCTs for the implementation of AI in health are not always ideal due to the implications of potential biases toward new technologies and/or issues with over-reliance (automation bias) (Tschandl et al. 2020). Moreover, the blurred lines between research and practise are likely to produce untraceable self-fulfilling outcomes that will not be detectable by focusing on accuracy, since false positives will appear as true positives (Mertens 2024). In the case of SERP, an RCT may especially not be feasible due to the way clinical judgement interacts with the triaging process, further complicating the blinded arm of the trial. Other implementation strategies are being considered such as ongoing evaluation-type studies to account for all variables.

This paper will use the SERP case study, described below, to illustrate how to analyse risk and assess potential benefits of new AI tools; where an RCT may not be feasible, nor the most effective way to evaluate a tool’s real-world capabilities. The proposed implementation of SERP will first be evaluated followed by an evaluation of risk more generally. This evaluation of risk will be undertaken in a way that is sensitive to ethical principles that apply for ongoing evaluation-type research, such as learning health systems (LHS), in so far as components of this framework applies to the proposed implementation strategy for SERP.

The issues identified in this paper also raise important questions concerning AI exceptionalism—the notion that AI poses exceptional risks that are fundamentally different from previous technologies thus requiring unique ethical considerations. These will be explored in relation to existing risks in ED, such as the emotional responses of triage nurses in a high-stakes environment. Then, a risk–benefit analysis will illuminate practical concerns around implementing new AI tools in healthcare, especially considering well-recognised human biases toward new technologies where end-users may either be wary of new tools or rely too heavily upon them, in each case leading to the potential misuse of the tool.

## Discussion

### Case Study: Score for Emergency Risk Prediction—Machine Learning Triage Tool for Estimating Mortality on Presentation at the Emergency Department (Xie et al. 2021; Wong 2024)

Emergency department (ED) triage includes an assessment of vital signs (body, temperature, pulse rate, respiratory rate, and systolic and diastolic blood pressure) as well as clinical history and presentation. Assessment is also reliant on the subjective judgement of healthcare professionals who should be attentive to ‘red flags’ such as patient disorientation or confusion, lethargy, severe pain, or distress.

In Singapore, all EDs currently use the national triage system known as the Patient Acuity Category Scale (PACS). PACS uses a symptom-based differential diagnosis approach, based on patients’ presenting complaints and objective assessments such as vital signs and the Glasgow Coma Scale.

On this scale there are four categories:

- P1: life threatening and requires immediate attention (most severe category)
- P2: in serious but stable condition, requiring emergency care
- P3: presents with mild to moderate symptoms and are stable to wait
- P4: non-emergency cases more appropriately managed in the primary care or other settings

Duke-NUS, a graduate medical school in Singapore, recently developed a new interpretable AI model to profile the mortality risk on presentation at the ED. This tool uses a machine learning model called Autoscore to produce a triage outcome score known as the Score for Emergency Risk Prediction (SERP) (Xie et al. 2020, 2023). SERP is an additive, point-based scoring tool, which makes it quick to calculate, easier to explain and easier to interpret.

Note that, unlike PACS, SERP only provides a raw risk score—an initial predictive output based on an algorithm to help healthcare professionals quickly identify high-risk patients. It does not instigate or determine a care pathway as it is not a ‘severity classification index’ which categorises patients into more broad severity levels such as those described by the PACS categories from 1 to 4 above. A severity classification index also usually involves interpretation of data and incorporates clinical judgement, and PACS is no exception. Raw risk scores, such as SERP, are therefore meant to inform but not determine distinctive care pathways as with a severity classification index.

A retrospective cohort study examined ED visits at Singapore General Hospital between January 2009 and December 2016 using the hospital’s Electronic Health Records. This study examined SERP scores for 224,666 patients in the model training cohort and 42,676 patients in the testing cohort.

The analysis showed that SERP had better prediction scores for mortality risk at 30 days than existing, commonly applied clinical triage scores, including PACS. Additional evidence from a retrospective study in Korea also supports SERP’s effectiveness. The Korean study aimed to externally validate SERP against other conventional scores, including the Korean Triage Acuity Scale (KTAS), and also found the performance of SERP to be superior to other scores for in-hospital and 30-day mortality prediction (Yu et al. 2022). Finally, a recent study addressed a class imbalance found in the original dataset which improved score performance for SERP + (Look et al. 2024).

However, it is currently unknown whether SERP can improve outcomes in actual clinical practise as further evidence is needed to validate its real-world predictive capabilities. Therefore, the research team has proposed a six-month prospective and non-interventional ‘silent trial’ whereby SERP and PACS would run alongside one another, but the SERP score would remain unknown to the triage nurse and not be used in practise. If the prospective data from the silent trial is successful and aligns with retrospective analysis, PACS would be replaced by a new model called ‘PAC + model 1’ where the SERP score would be used to finetune the PACS score in practise.^1^

A SERP score of 26/59 was found in one study to be the optimal threshold that maximises the sensitivity and specificity for predicting 30-day mortality. ‘Sensitivity’, sometimes called the ‘true positive rate’, refers to the proportion of people with a condition who test positive and whose cases are detected. ‘Specificity’, sometimes called the ‘true negative rate’ refers to the proportion of people without a condition who receive a negative result. Adopting this score as a threshold, nurses would be able to finetune the PACS score as follows:

If the SERP score is < 26/59, the tool would indicate that the patient should be down-triaged by 1 PACS category (e.g. from P2 to P3). If the SERP score is ≥ 26/59, the tool would indicate that the patient should be up-triaged by 1 PACS category. The final decision to finetune in this way, however, will still be one determined by clinical judgement.

This bi-directional approach would allow the PACS score to be calculated and applied as normal, and the SERP score to be calculated and given to the healthcare professional as additional information for adjusting triage as appropriate.

## Is the Proposed Evaluation Model Appropriate?

As AI models are sociotechnical in nature, it is recommended to test AI in clinical practise rather than relying solely on accuracy and data analysis. This suggests the need for pre-implementation evaluation via something like an RCT (Carter et al. 2020; Elish 2018). SERP’s retrospective accuracy only demonstrates efficaciousness under controlled conditions and does not imply necessary efficacy in the real world. The same holds in the case of a successful RCT where new issues may arise during implementation despite the data evidenced.

However, an RCT in this case may not produce the data necessary to evaluate SERP for several reasons: (1) the nature of the triaging process is such that the clinical judgement of nurses plays an integral role in determining the PACS score; (2) mortality risk is only one piece of the triaging puzzle (i.e. it does not give the full picture for determining risk and/or success of patient outcomes), (3) it is highly challenging to deploy a traditional RCT in the emergency setting, as randomisation, obtaining informed consent may not be feasible, and (4) the RCT itself may introduce added delays and bias to the triage process, which may adversely affect patient outcomes.

Regarding the first reason, current PACS scores are determined based on the expertise of the triage nurse after specialised training. The final score is ultimately based on clinical judgement and highly dependent on the experience of the nurse, and so the blind arm of an RCT would fail to account for how nurses employ SERP in practise.

Regarding the second reason, if a head-to-head RCT design were put forward, one group of participants would receive care based on the PACS score and another based on the SERP score. Since SERP only provides key information about the prediction of mortality risk, it does not encompass the complexities of variables that go into the final clinical judgement, and so this design would fail to accurately depict SERP’s ability to better patient outcomes.

A PACS score is also designed to inform a course of clinical action directly. The impact of clinical care decisions based on the PACS score is likely to affect the patient’s eventual outcome in self-defeating or self-fulfilling ways. Knowing retrospectively what the outcome would have been counterfactually is difficult and remains uncertain, especially in cases of self-fulfilment where false positives appear as true positives (Mertens 2024). This complicates research integrity and quality assurance.

In contrast, the PAC + model 1 involves SERP supplementing PACS as the baseline, providing enhanced risk stratification backed by mortality prediction. The risk scale would remain 1–4 which would create minimal disruptions to workflow, and the triage nurse would still be at liberty to use his/her clinical judgement to override the score based on clinical experience and expertise. This is important as PACS already provides clear clinical management plans associated with each triage score that, despite inevitably obscuring counterfactual information, have been demonstrated as sufficient and effective over time. Therefore, if a different plan to fully implement SERP was under consideration, this would need a rigorous approach and a stronger evidence base to establish new clinical pathways as there is currently no known real-world relationship between the raw risk score produced by SERP and requisite ED workflow processes that respond to the degree of risk identified.

Regarding the third reason, it is highly challenging to deploy a traditional RCT involving individual patient-level randomisation, in the emergency setting, as randomisation, obtaining informed consent may not be feasible. In Singapore, regulations around clinical trials in emergency situations only allow for waiver of informed consent with certification by two senior physicians, which is not feasible in the ED environment. Also, the RCT itself may introduce added delays to the triage process, due to the randomisation and consent procedures, which may adversely affect patient outcomes. Finally, inclusion and exclusion criteria for such a trial, bias introduced during consent (sicker patients could be expected to be less likely to consent), plus likely non-compliance and patient selection by clinical staff would introduce selection bias that may confound any interpretation of the trial results.

Finally, while we should be cautious about implementing SERP too soon considering the lack of prospective data, delaying SERP’s implementation could also have negative consequences by failing to address urgent and ongoing inefficiencies in EDs, so getting the timing right is crucial. Hence, alternative study designs, such as hybrid implementation and evaluation type studies, are more feasible in this setting rather than a head-to-head RCT.

As an alternative to an RCT, McCradden et al. argue for a three-part research ethics framework similar to the proposed framework for SERP. The three stages flow as follows: (1) exploratory, hypothesis-generating data access; (2) silent period evaluation; (3) prospective clinical evaluation (2022). This is in order to move beyond validation through technical performance in historical datasets of ML tools and account for their performance in clinical practise in an ethical way, especially in consideration of biases on the part of healthcare professionals towards new tech. ‘The basic ethical challenge’, they argue, ‘is that the addition of an ML model to an extant clinical workflow may involve significant departures from the standard of care, with attendant risks.’ In the case of SERP, these considerations are essential, making the silent trial the most appropriate first step and PAC + 1 an appropriate move forward, but the ethical implications must still be considered.

## Frameworks for Ethical Implementation: Ongoing Improvement and Learning

Various approaches to clinical implementation of new devices and processes, including AI, focus on systematically introducing a new device or process, embedded in a cycle of iterative evaluation of performance, safety, and efficacy and a philosophy of continuous learning. Well-recognised approaches include quality improvement (QI), quality management systems (QMS), and learning health systems (LHS) which already have a grounding in the Singaporean health system.

The SingHealth Duke-NUS Institute for Patient Safety & Quality (IPSQ) defines QI in healthcare as a systematic approach ‘making processes safe, efficient, patient-centred, timely, effective and equitable’ considering the complexities of the healthcare environment (SingHealth Duke-NUS Institute for Patient Safety & Quality 2020). Unlike research, QI must not be conducted to generate evidence to support an intervention’s efficacy, but it can involve evaluating and changing practise (Provost and Murray 2011). In QI, generally the focus is on system functioning and change rather than the individual behaviour or response.

Singapore’s Health Sciences Authority (HSA) endorses a quality management system (QMS) in the management of software medical devices by referring to the International Organization for Standardization (ISO) requirements. This may include designing, testing, and implementing changes using real time measurement for improvement. QMS principles include leadership and organisation, life cycle supported processes, and product realisation activities including risk assessment, hazard analysis, and risk mitigation (Regulatory Guidelines for Software Medical Devices—a Life Cycle Approach Revision 2.0 2022). HSA thus proposes a three-step approach for systematic risk management: (i) identify all possible hazards, (ii) assess the associated risks, (iii) implement mitigations or controls to reduce risks to acceptable level, and (iv) observe and evaluate effectiveness of mitigation measures (18).

The Singapore Ministry of Health’s Artificial Intelligence in Healthcare Guidelines AIHGIe refers to IMDRF to define implementation criteria to ensure actual clinical outcomes of AI-MD are measured and assessed. The IMDRF also notes that management of quality should be an interactive and continuous process (See Annex 1).

While dominant research ethics paradigms make a sharp distinction between research and practise, learning health systems (LHS) offer a way to integrate them. Examples include QI and comparative effectiveness research. An LHS differs from QI and QMS in that it moves beyond an implementation framework and into the ethical realm. For this reason, LHS offers the support for the claims in this paper as will be outlined in the subsequent sections.

LHS suggests the following principles as guidance for an ethical framework:

1. Respect the rights and dignity of patients
2. Respect clinician judgements
3. Provide optimal clinical care to each patient
4. Avoid imposing nonclinical risks and burdens on patients
5. Address health inequities
6. Conduct continuous learning activities that improve the quality of care and health systems (Faden et al. 2013)

Considering the risks and benefits posed by SERP, safe and effective implementation into clinical practise can be appropriately managed through consideration of such principles.

In what follows, we may draw on the LHS framework as grounding for the ethical approach in this paper. However, any one framework or principle need not constrain how to manage individual cases. Certain principles may not apply or may need to be challenged so that there is flexibility to accommodate the complexities of each case, thus making obligations case dependent. A bottom-up approach to applying said frameworks takes the unique ethical considerations of each case as the driver of the salient principles we can draw upon from QI, QMS, and LHS.

While these frameworks do not serve as validation for the current analysis from a top-down perspective, the particularities from the SERP case guide the evaluation of the implementation plan and draw from these frameworks as grounding. The risk analysis and mitigation strategies outlined below will demonstrate this approach as they align with the aims and practises found in these paradigms.

## Assessing and Mitigating Risk in a Proposed Implementation Model

The LHS principles above highlight the ethically salient reasons to be concerned with the assessment of risk in implementing SERP. In particular, the requirement to provide optimal clinical care shapes how the distinctive risks associated with using SERP in real-world settings should be determined. In line with the bottom-up approach in this paper, in this section, each LHS principle is applied insofar as it is useful in the context of the risk being addressed. In this way, we adopt a *principle-sensitive* approach that makes use of each principle when it is relevant and applicable in the context of the case scenario to address the specific ethical issues identified.

### AI Exceptionalism

AI risk should be ethically evaluated similarly to other medical interventions and processes appropriate to the level of risk they pose. The assessment of risk needs to be calibrated to levels of risk that are acceptable for translation of any new tech or other interventions into clinical contexts, and not necessarily treated as an exceptional type of risk.

AIHGIe states that the decision to implement AI medical devices should involve a risk assessment and defines risk as follows:

Risk is defined as a function of (a) impact and (b) likelihood; (a) Impact—severity of patient harm if AI-MD is erroneous, and how quickly errors could be discovered and rectified; (b) likelihood—probability for errors to occur depending on the AI-MD model and level of human oversight. (See Annex 2)

As we lack the prospective data to accurately identify real-world risks of implementing SERP, it is difficult to measure SERP’s risk level under this definition. SERP has proved unlikely to make errors based on the results from the retrospective studies where it had better predictive performance than existing scores with an AUC of 0.874 to 0.905 (Look et al. 2024). Should the silent trial be successful and we move to PAC + model 1, we can infer that the severity of patient harm would be low if SERP’s conclusions are erroneous, because the PACS score would be applied as normal and there will always be oversight by a nurse who will make the final triage judgement. It is also relatively unlikely that SERP will make additional errors beyond those already occurring in EDs operating with only PACS. At the very worst, it may invisibly exacerbate existing mistakes that are hard to catch in current practise. In any case, SERP is likely to improve upon the existing PACS scores by offering more information to aid nurses in correctly triaging patients.

The subsequent question is whether we have sufficient evidence and reason to justify translating SERP into practise via the PAC + model 1, such that we are satisfied that the potential benefits outweigh the risks. Reasonable risk refers to the proportionality of the risk compared to the benefits, where the risk correlates to the potential harm induced and the benefits to the utility of the outcomes The potential benefits would have to be proportionate to the potential risks of implementing SERP where a reasonable level of risk may be justifiable considering the risks already present in the real world (i.e. healthcare professionals making decisions in a high-pressure environment, fallibility of traditional scoring systems, etc.).

The central risk associated with SERP is the real-world implications of false negatives and false positives and their clinical correlates, positive and negative predictive values. False negatives happen when SERP incorrectly underestimates mortality risks, informing clinical decisions that fail to attenuate risk of death or serious harm correctly. This may lead to worsening patient outcomes by inappropriately putting high-priority patients in a lower triage category. False positives occur when SERP incorrectly overestimates mortality risks which can lead to the unnecessary use of limited medical resources when patients who are not in as urgent need of care are prioritised and may divert attention away from those with more urgent needs. Note that false negative risk accrues to the patient who is given a false negative whereas false positive risk accrues to other patients, who are crowded out.

Most important to the evaluation of a predictive algorithm, and thus of SERP, is the difference in obtainable quality assurance between the two false risk predictions. In the case of a false negative, a patient mistakenly identified as low risk, will be recognised as suddenly needing more care than envisioned. In contrast, a false positive involving a patient falsely identified as too high a mortality risk to be given limited resources in comparison to more salvageable patients, will go unnoticed as the withholding of treatment will result in their ‘predicted’ demise. Essentially, false negatives can later be identified as false, whereas false positives will not be recognised as false, as the self-fulfilling outcome resulting from the prediction will vindicate that prediction (Mertens 2024; Mertens et al. 2022). In general, considering the risk of self-fulfilling prophecies, checking the algorithm for accuracy will be insufficient to provide quality assurance (Mertens et al. 2022; King and Mertens 2023).

In evaluating an appropriate risk threshold, we must understand if these risks are worth the benefit SERP could potentially bring to EDs. In the following sections, we will consider the relevant risks already at play in emergency medicine and evaluate whether SERP, and risks posed by AI more generally, should be thought of as exceptional. As the evaluation of any new intervention (involving AI or not) involves some degree of risk, and since we may not always be able to remove risk completely, ethical concerns should focus on minimising risk and assessing the threshold of acceptable risk.

### Technology Bias and Automation Bias

Healthcare providers’ biases may lead to a violation of the LHS principles of ‘conducting continuous learning activities that improve the quality of care and health systems’ and ‘avoiding imposing minimal nonclinical risk or burden on patients and families’ when bias interferes with the proper evaluation of prospective data collected in the silent trial and PAC + model 1.

Here, it is important to clarify first what biases are in play. Technology bias refers to resistance to new technologies either due to fear that it is too new to trust, or pressure to conform to the status quo, or both. Conversely, automation bias refers to the overreliance on AI that can occur when an AI tool has proven efficient, causing humans to lose critical consideration for the AI conclusions (Parasuraman and Manzey 2010). These are well documented phenomena in healthcare and beyond (GE Healthcare 2023; Georgiana Juravle et al. 2020; Global Data Thematic Intelligence 2023; Chugunova and Sele 2022).

Both technology bias and automation bias threaten our capacity to effectively quantify the performance of clinical AI independent of human users, thereby creating a barrier to determining the potential benefits of AI tools. Moreover, technology bias and automation bias can distort the correct use of the AI tool by clinicians, possibly resulting in a threat to the health of the patient if the tool is used incorrectly. Therefore, caution should be practised in adopting new technologies such that awareness of the underlying reasons for caution fairly reflect the level of potential risk.

Potential biases could manipulate the ‘natural’ data in PAC + model 1, thus making it difficult to effectively measure SERP’s actual performance should these biases cause users to inappropriately apply its scores. If a healthcare professional approaches SERP with resistance or overreliance toward its output, then we cannot correctly evaluate SERP’s accuracy.

To avoid the risk of imposed bias, an alternative to applying SERP as proposed in PACS + model 1 would be to make the finetuning mandatory and not allow nurses to make the final judgement. This runs the risk of failing to respect clinical judgement, an LHS principle. It would also mean a significant deviation in workflow that could result in misuse. Since the most important and desired information is whether or not SERP improves patient outcomes, and these biases are of real concern; it is important to collect data that includes how they might affect SERP’s real-world capabilities. The PAC + model 1 should be successful in accounting for this variable and thus necessary to appropriately understand real-world outcomes.

PAC + model 1 drives forward the ‘continuous learning’ and ‘avoiding imposing minimal nonclinical risk’ as much of the same data already being collected for the PACS score can be reused. A few additional questions, such as history of cancer diagnosis, will take less than five minutes of patient time.

The silent trial also invokes the ‘continuous learning’ principle by first validating the performance of SERP with prospective data, maintaining the likelihood that there will be less of an impact due to the risk of false negatives by continuing to collect prospective data that will avoid manipulation by end-users. It also maintains respect for clinical judgement and hopefully keeps the data as ‘natural’ as possible until it is prospectively validated.

Finally, retrospective comparative studies can help avoid SERP’s further exacerbation of missed false positives in the crowded out patient group with the highest mortality risk. These patients end up not receiving care when the care could have helped, in contrast to what the risk score predicted. When comparing the size of this patient group in the PAC + model 1 with the one in PACS, an increase in size is likely to reflect an increase in missed false positives.

### Influence of Emotional Responses

Alongside PACS are various other frameworks used for assessing risk such as the Canadian Emergency Department Triage and Acuity Scale (CTAS), Emergency Severity Index (ESI), Manchester Triage System (MTS), Australasian Triage Scale, and Korean Triage and Acuity Scale (KTAS) (Christ et al. 2010; Yu et al. 2022). Each scale uses similar metrics (vital signs, healthcare professional judgement, etc.) and evaluates risk on a severity scale of four or five. However, different scales may focus on different aspects of severity. For example, ESI incorporates resource needs in the triage ratings, whereas PACS triage is based on presenting symptoms and objective clinical data (Fong et al. 2018). The Australasian Triage Scale, on the other hand, focuses on the time a patient can safely wait (Yancey and O’Rourke 2021).

Each system runs the risk of under-triaging (false negatives) or over-triaging (false positives). This becomes an ethical issue when the interaction between a score and the exercise of clinical judgement results in a false negative or positive, especially when the clinician’s judgement is influenced by emotional or environmental factors. In such a situation, the heightened risk of patients being triaged incorrectly may cause serious (and arguably avoidable) harm depending on the medical severity of the situation (Grossmann et al. 2012).

In the instance of overcrowding, for example, patients may be assigned a higher score (indicating higher priority) due to subconscious pressure put on the healthcare professionals to free up space in the waiting room. While these decisions are often well-intended, they may lead to inefficiencies in the workflow in the best case, and otherwise preventable deaths in the worst.

As with the possibility of false negatives and false positives that could occur due to a SERP failure, current scoring systems as well as healthcare professionals are prone to producing similar mistakes. These kinds of mistakes may be due to the high-pressure nature of the role in ED, susceptibility to emotional responses, and environmental distractors such as noise and task interruptions—obstacles which are inherent to emergency care and which AI cannot plausible be expected to overcome (Gorick 2022; Delmas et al. 2020).^,^

On the one hand, emotions can be incredibly helpful to clinicians in making ethical decisions by invoking empathy, care, and compassion. On the other hand, healthcare professionals may need to compartmentalise their emotions in order to avoid rash decision-making that may deviate from standards set by institutional and regulatory frameworks (Almeida et al 2023). In emergency medicine, it is particularly difficult to keep judgements impartial in the face of suffering patients and urgent requirements. While AI’s lack of emotion may help to keep decision-making less emotionally charged, it can be argued that this will not always produce the best outcome. Therefore, the emotional responses of healthcare professionals and the lack of emotion in AI can be said to pose comparable risk.

### Interpretability

Interpretability in AI seeks to develop tools that transparently allow humans to understand the results and output created by algorithms. This understanding does not necessarily require any technical knowledge on the part of the user, but rather a high-level grasp of how the tool works and comes to its conclusions. This is unlike black box models which create predictions that are too complicated for human understanding thus creating barriers to adoption (Yun et al. 2021).

While interpretability cannot be strictly defined, ‘an interpretable machine learning model is constrained in model form so that it is either useful to someone, or obeys structural knowledge of the domain, such as monotonicity, causality, structural (generative) constraints, additivity, or physical constraints that come from domain knowledge’ (Rudin 2019). In other words, the tool provides its own domain-specific explanation such that the end-user can grasp the processing of inputs and resulting outputs (Yohei et al. 2023).

The potential risks of non-interpretable black box models can have severe consequences due to a lack of transparency and accountability. Such tools have led to the release of dangerous criminals on bail (Wexler 2017) and poor use of limited valuable resources (Varshney and Alemzadeh 2017). An interpretable model poses far less risk than black box models by giving users the opportunity to use their own judgement and expertise in evaluating the quality of the tool’s output.

Thus, interpretability results in clinicians’ higher assurance in the validity of conclusions and therefore safety of the tool insofar as it may help to reduce underlying concerns they may have surrounding new tech. That is, interpretability may not necessarily describe anything about the riskiness of a tool itself, but the more interpretable a tool is, the more comfortable the user will probably be trusting it and thus be more likely to use it correctly, and this lowers the overall risk of using the tool. The prerequisite is, of course, that the user has the required expertise and that the interpretability is made transparent to the user.

Interpretability also helps to ensure the user will appropriately employ the tool and not impose his or her own biases, whether positive or negative (Khairat et al. 2018). For these reasons among others, it has been argued that models that are explainable should be required in the clinical setting (Yohei et al. 2023)). Further arguments also support that the way forward in designing models is to ensure interpretability in the first place rather than try to explain black box models post-hoc (Rudin 2019).

SERP is an interpretable model. The SERP scoring models are derived from AutoScore, a machine learning point-based clinical score generation algorithm. The input of six variables makes up the raw risk score: age, heart rate, respiratory rate, systolic and diastolic blood pressure, and a patients’ cancer history. This makes it clear to the care team why some patients are given higher scores than others. In comparison to other complex models, point-based scores also prove more explainable by enabling users to easily build interpretable clinical scores. These scores can then be implemented and validated in clinical practise (Xie et al. 2021). SERP’s interpretability thus makes it less risky by increasing the chance that nurses will use it correctly.

### Asymmetrical Risk Mitigation Strategy

A further asymmetric risk strategy called the PAC + model 2 has also been proposed, adopting a more cautious approach whereby the finetuning only goes in one direction, up-triaging patients with high mortality risk, while patients with low mortality risk remain in the same triage level (Wong 2024). The worry with this strategy lies in the potential violation of the LHS principle of ‘addressing health inequities’ by prioritising PAC level 1 patients on the basis of unsound precautionary reasons.

PAC + model 2 effectively avoids an increased risk of false negatives, though not of false positives. On this model, if the SERP score ≥ 26, the tool would indicate that the patient should be up-triaged by 1 PACS score. If the SERP score is < 26, the tool would indicate that no change to the PACS score should be made. Again, the final decision to finetune will be one of clinical judgement.

Further retrospective data has shown this model to have slightly lower predictive capabilities than PAC + 1 for 30-day mortality: PAC + model 1 AUC 0.828 [95% CI 0.820–0.836]) and PAC + model 2 (AUC 0.812 [95% CI 0.805–0.818]. However, both are superior compared to the reference model PAC (AUC 0.722 [95% CI 0.714–0.719]) (Wong 2024).

The important factor is how this will affect overcrowding. While the PAC + model 1 model resulted in a significant increase in the percentage of patients triaged to lower priority levels 3–4 compared to PAC alone, the PAC + model 2 significantly increased the number of patients triaged to priority level 1 compared to PAC + model 1 and PAC. With the potential for overcrowding to negatively impact patient outcomes due to hindering healthcare professionals’ ability to provide safe and optimal care, caution toward the PAC + model 2 model is warranted.

The worry is that the PAC + model 2 model could result in negative effects for those who are crowded out due to an increase in priority 1 patients. With limited medical resources, over-triage can have detrimental effects. On the one hand, it results in by diverting attention away from those who need more urgent care than what their triage score provides them. On the other hand, the more patients are crowded out of care, the greater the risk of more false positive cases being missed. This could result in widening health inequalities making the risk to patients, current and future, too high.

Instead, PAC + model 1 upholds the LHS principle of ‘providing optimal clinical care’ by the outcome potentially significantly decreasing wait times and improving accuracy for mortality risk. This principle also points to the need to ensure alignment of the SERP threshold score within the PAC + model 1 to clinical governance requirements and ongoing clinical review of identified real-world application. For example, if local clinical guidelines demanded sensitivity to be prioritised over specificity, then, the SERP threshold score for recommending a modification to the PAC score would need to be amended prior to implementation. Depending on the day and institution, median wait times for admission in Singaporean hospitals can range from one hour to over 28 h making SERP especially impactful.

## Conclusion

The potential benefits of SERP could greatly enhance the efficiency of triage process leading to reduced delays, decreased overcrowding, and more effective patient prioritisation. Since SERP is a clinical decision support tool and not a decision-making tool, the risk of patient harm is lower compared to systems that directly dictate patient care pathways. While concerns about technology and automation bias are valid, they do not represent an exceptional risk beyond those already present in emergency departments. Moreover, the interpretability of SERP reduces the likelihood of misuse by humans. Therefore, implementing SERP in such a way that looks beyond RCT-based methods offers a meaningful example of how to assess and address ethical and efficacy issues of these new technologies. The current implementation strategy beginning with a silent trial followed by the observational-style model of PACS + model 1 outweigh the risks, and aligns with LHS ethical principles.

The overarchingly salient ethical strategy appears in the LHS principle of ‘conducting continuous learning activities that improve the quality of care and health systems’. Even if SERP is successful over time, EDs should constantly monitor effectiveness and safety. Baseline performance that reflects the minimum level of performance SERP should surpass to be considered valuable should be understood to evaluate whether SERP is indeed contributing to bettering patient outcomes. Retrospective studies comparing SERP implementation with PACS alone can furthermore check for increased rates of untraceable false positive rates. These continuous learning activities require collaboration among all stakeholders including the developers, clinician-scientists, IRBs, and end-users. The ethical principles of LHS provide an appropriate framework to guide the introduction of clinical AI tools such as SERP because they incorporate and integrate values from both clinical and research ethics. By comparison, traditional research ethics, and especially RCTs, do not necessarily provide the best, safest, and most ethical validation method for clinical AI tools.

## Annex 1

MOH AIHGIe refers to IMDRF to define implementation criteria to ensure actual clinical outcomes of AI-MD are measured and assessed. ‘Clinical association of the outcomes of an AI-MD to its intended use can be established through existing evidence (e.g. literature, original clinical research, guidelines), or generating new evidence (e.g. data analysis, clinical trials)’ (p. 23).

IMDRF categorizes impact, which influences risk, based on how it interacts with clinical judgment in three ways: (1) treat or diagnose, (2) drive clinical management, and (3) inform clinical management. Risk is then defined based on the state of the healthcare situation or condition on three levels: (1) non-serious, (2) serious, and (3) critical. See p.14: https://www.imdrf.org/sites/default/files/docs/imdrf/final/technical/imdrf-tech-140918-samd-framework-risk-categorization-141013.pdf

## Annex 2

Singapore’s AI-MD validation scheme in the MOH AIHGIe. 4.10.1 Developers should periodically evaluate and validate their AI-MD’s performance to ensure it minimally meets the clinical practice baseline (see Section 4.4), and verify the accuracy, and reproducibility of the AIMD’s algorithmic decisions. 4.10.2 The clinical performance of an AI-MD involves more than just technical measures of its algorithm’s performance (e.g. area under the curve (AUC)—receiving operating characteristic curve (ROC) or precision-recall curve (PRC), true positive rate, positive/negative predictive value, Cohen’s Kappa score). Developers should work with implementers to ensure that the actual clinical outcomes of the AI-MD (i.e. impact on patients when the AI-MD is introduced to their care) are measured and assessed. 4.10.3 Table 3 sets out a suggested stepwise AI-MD validation approach, and the types of risks it assesses. Developers should compare results from each step with the current clinical practice baseline (23).

Footnotes in the document state: 15: Outcome-based performance variables should be considered if the outputs of the AI-MD are not directly measurable (i.e. classifiable outcomes, performance scores). Examples of such AI-MDs include those that are usually designed for clinical interventions (e.g. surgical AI robots, AI-based cancer treatment). 16: It has become commonplace to evaluate machine learning algorithms based on overall measures like accuracy or area under the curve (AUC). However, one evaluation metric may not always capture the complexity of performance. As an extreme illustration, an algorithm designed to predict a rare condition found in only 1% of a population can be extremely accurate by labelling all individuals as not having the condition. This tool is 99% accurate, but completely useless. Yet, it may ‘outperform’ other algorithms if accuracy is considered in isolation—STAT Report (2021): Promise and Peril—How artificial intelligence is transforming healthcare. 17: Ability of an AI to cope with and operate correctly (as a system or components of it) in the presence of invalid/erroneous inputs or stressful environment conditions (e.g. high volume of inputs, adversarial attacks on AI models) (24).

## Footnotes

1 If the risk score is above a threshold, then, the prediction is high risk; if it is below a threshold, it is low risk. The threshold may change due to its use context and thus requires localised calibration. The threshold determines sensitivity and specificity where the ‘best’ threshold will be the one that gives a good trade-off between sensitivity and specificity. For the purposes of this paper, we will use a study where the SERP score was programmed on a scale from 1 to 59, following the research report, with a score of 26/59 identified as being the optimal threshold that maximises the sensitivity and specificity for predicting 30-day mortality. It should be noted that the score can be re-scaled, thus increasing the threshold figure.
